# Prevalence and comorbidity in a Swedish adolescent community sample – gambling, gaming, substance use, and other psychiatric disorders

**DOI:** 10.1186/s12888-022-04218-1

**Published:** 2022-09-06

**Authors:** Arne Gerdner, Anders Håkansson

**Affiliations:** 1 School of Health and Welfare, Department of Social Work, Jönköping University, Box 1026, 551 11 Jönköping, Sweden; 2 Department of Medical Sciences Lund, Lund University, Psychiatry, Lund, Sweden

**Keywords:** Substance use disorder, Gambling disorder, Internet gaming disorder, Psychiatric disorder, Prevalence, Comorbidity, Adolescent, Community sample, Clinical interview

## Abstract

**Background:**

This study investigates a broad spectrum of psychiatric disorders, substance use disorders, gambling, and internet gaming disorders in Swedish 18-year-old boys and girls with the aim of estimating the prevalence of disorders and comorbidity.

**Methods:**

We used a two-phase design with screening to detect candidates for clinical interviews. Screening included 949 adolescents (55.6% girls), out of which 758 adolescents (57.0% girls) were selected for interview with at least one of four instruments: M.I.N.I., ADDIS, NODS and IGDS. Of these, 387 (61.2% girls) were interviewed. Gender separated prevalence was estimated on the assumption that those selected but not interviewed had the same distribution as those interviewed based on similar outcomes above screening cut-offs. Comorbidity between types of disorders was estimated on similar assumptions. In addition, comorbidity between dyads of the ten most common specified disorders was calculated based on recorded data without these assumptions.

**Results:**

We estimated that 14.6% met the criteria of a substance use disorder (SUD), mostly concerning alcohol and more frequent in girls than in boys. Those meeting the criteria lifetime of at least one of 16 other psychiatric disorders were 26.7%, more than twice as frequent in girls compared to boys, and with depression being the most common disorder. Gambling and gaming disorders were found almost exclusively in boys, of which 5.8% met the criteria for gambling, and 2.3% for gaming disorders. Of girls with a SUD, 40% also had a psychiatric disorder, while on the other hand more than 28% of girls with a psychiatric disorder also had a SUD. In boys with a SUD, 22% had another psychiatric disorder, while 15% of those with a psychiatric disorder also had a SUD.

**Conclusions:**

Psychiatric comorbidity is common in SUDs in adolescents, which calls for screening and diagnostic efforts in young patients presenting with symptoms of SUDs. Girls with SUDs are at higher risk of also suffering from psychiatric conditions. Gambling and gaming disorders appear in a substantial minority of adolescents and warrant further study of their comorbidity. Since prevalences and comorbidity were estimated on the assumptions mentioned, some caution in interpreting the results is needed.

## Background

The mean global prevalence of mental disorders in children and adolescents (5–17 years) was recently estimated to be 13% [1]. This estimation results from the Global Burden of Disease (GBS), an international research programme building on national reports with a mean global coverage of about 6.7% of the world population [2]. A meta-analysis of 41 epidemiological studies using diagnostic interviews based on International Statistical Classification of Diseases and Related Health Problems (ICD) or Diagnostic and Statistical Manual of Mental Disorders (DSM) gave a similar estimation (13.4%) of the worldwide-pooled prevalence of mental disorders in children and adolescents (6–18 years) [3].

In the GBS [1], Sweden was represented with only two studies: one on attention deficit hyperactivity disorder (ADHD) based on clinical registers [4] and one on autism spectrum disorder based on clinical interviews [5]. The meta-analysis included only studies assessing three or more psychiatric problems and found no Swedish study meeting that criterion [3]. A systematic review found 15 community studies using systematic diagnostic instruments to assess psychiatric disorders in adolescents, including 10 that also assessed substance use disorders in the same rigorous way [6]. None of these were European. A recent review confirmed the lack of Swedish adolescent psychiatric epidemiology studies [7]. This study therefore concerns psychiatric epidemiology among Swedish adolescents, estimating the prevalence and comorbidity of psychiatric disorders, including substance use disorders (SUDs) as well as gambling and gaming problems.

Cederblad reported on 50 years of child and adolescent psychiatry epidemiological research in Sweden [8]. There are many studies on emotional and behavioural problems in terms of internalising and externalising, but these do not estimate the prevalence of specific disorders. There are more recent studies on psychiatric problems based on screening instruments in community samples of children or adolescents, such as on social phobia (social anxiety) [9]; depression [10]; body image, depression, and anxiety [11]; and gambling problems in adolescent boys [12]. These and other screening studies are helpful for estimating the extent of various problems, but they are not equivalent to diagnostic studies based on the criteria of ICD or DSM. Although Sweden has long statistical series on adolescent drinking and drug use with national school surveys conducted since the 1970s [13], these do not include consequences necessary for a SUD diagnosis. There is, however, a study on alcohol dependence according to DSM-IV criteria in Swedish adults (17–84 years) [14], showing a general prevalence of 4% (men 4.8%, women 3.1%). The study also presented data on age groups, and in the youngest group (17–29 years), 7.1% of men and 7.7% of women were found to be dependent.

Neither of these studies can be used to estimate psychiatric comorbidity in adolescence, since they all, including the diagnostic studies mentioned, deal only with one (or two) psychiatric disorders each. Therefore, epidemiological studies covering a broad spectrum of adolescent SUD and mental health disorders based on structured diagnostic interviews are needed, since such studies are still absent in Sweden although repeatedly requested by governmental task forces [15, 16].

Often, prevalence studies are carried out based on healthcare records that cover a fraction of the problems due to the fact that many people do not seek care. Only about half of those affected by depression, for example, seek treatment [17, 18]. In Sweden, only visits to doctors in specialised clinics are recorded, not visits to primary care, and not visits to other health care professionals [7]. Population interview studies may be used to also include these persons, but such studies are extremely costly and may still suffer from large data loss due to problems in making contact with the persons or due to their unwillingness to participate [19]. A two-phase design is one way to make epidemiological interview studies more feasible and efficient [20]. This design depends on the use of a screening in phase 1, directed towards the population or towards a representative sample, to select those invited to structured interviews in phase 2. The screening tests in phase 1 need to have high sensitivity to include all relevant cases, and reasonably high specificity to exclude enough of those who can be assumed to be non-cases. The interview instruments need to have diagnostic validity based on established diagnostic criteria from ICD or DSM. However, two-phase studies are not without problems. There is a risk of losing persons selected for interviews, either due to unwillingness to participate in a more time-consuming interview, or simply because of problems in establishing contact. In that situation, there may be a need to address attrition bias and make estimates of probable cases among those lost to interview.

In addition to gaining knowledge on SUD prevalence, mental health disorder prevalence and their comorbidity, there is reason to introduce behavioural addictions to the screening and diagnostic procedures in adolescents. Gambling disorder has been recognised as an addictive disorder related to gambling for money. This condition has been well-established for decades, with a well-known symptomatology involving indebtedness, a failed attempt to reduce gambling despite severe consequences, and emotional and social consequences [21]. Despite this, the implementation of screening and treatment procedures in this condition has been variable and limited [22], and typically with low rates of treatment seeking [23]. More recently, as recognised in the diagnostic manual of the World Health Organization, a gaming disorder has also been recognised, describing an addictive and maladaptive pattern related to video games and similar gaming modalities [24]. Due to the relative novelty of behavioural addictions in clinical research and in policy making in many countries, general population studies of adolescents’ mental health, such as the present one, often may not include these behavioural addictions, especially not the most recently added gaming disorder diagnosis.

Adolescence as the phase of transition from being a child to an adult is roughly considered to be the period between 11 and 19 years of age, often divided in three stages – early adolescence (about 11–13 years), middle adolescence (about 14–17 years), and late adolescence (about 17–19 years) [25]. It is a period in life with a lot of physical, mental, and social strain that may contribute to addictive and other psychiatric disorders, with early onset peaking in mid adolescence [26]. A study on prevalence and comorbidity carried out in late adolescence should therefore have optimal possibilities to capture the situation.

The present article reports from a two-phase design study. The aim is to estimate the prevalence and comorbidity of SUDs and other psychiatric disorders, including gambling and gaming problems based on ICD or DSM criteria, in a Swedish community population in late adolescence. Expected attrition bias will be addressed.

## Methods

This study is part of a larger research project on development in adolescence from the age of 12 or 13 and with six data collection waves until the age of 18. LoRDIA (Longitudinal Research on Development In Adolescence) has data on mental health, use and misuse of alcohol and drugs, disability, peer and family relations, norm-breaking behaviours etc.

LoRDIA started in 2013 in four small and medium-sized municipalities (10,000–38,000 inhabitants) in southern Sweden. Two were characterised as industrial, and two as commuter municipalities. One commuter municipality was located in close proximity to a large city. The unemployment rate, annual income, educational level, and proportion of first-generation immigrants across the four municipalities were close to the national means [27]. All children in two school-year cohorts were invited to participate: those who in autumn 2013 were in grades 6 and 7 and approximately 12 and 13 years old. Out of all 2150 invited, 1885 (88%) agreed to participate with parental acceptance and they constitute the LoRDIA study population. Those who declined participation showed no differences in demography (gender and immigration status) or school performance (merit rating and attendance) [28].

Comprehensive data collections were conducted annually in three or four waves for the respective cohorts up to grade 9 at about the age of 15. During these years, the data collection had a high turn-out, with 96% participating at least once and 70–85% on each occasion. Wave 5 questionnaires were collected in the autumns of 2017 and 2018, respectively, when the two cohorts were in their second year of upper secondary school, approximately 17 years old. In Wave 5, about half (i.e., 50.3%) of the original study population participated.

Wave 6 is the data collection in focus here and was carried out when the adolescents were 18 years old. It was conducted as telephone interviews in the evenings, after school, by 20 trained interviewers with professional skills in psychology, or psychiatric nursing. In addition to professional merits, all 20 interviewers were trained by the authors and were passed as qualified for the task.

Wave 6 depended on having participated in Wave 5. At the end of the Wave 5 questionnaire, information was provided about the planned interview and contact addresses (telephone and e-mail) were requested. Of the 949 Wave 5 participants, 95% provided such, thereby showing some preparedness for participating in interviews. Since it would not have been feasible to interview all, Wave 5 also included screening instruments. Only those who scored above the chosen cut-offs were invited to interviews. Concerning ethical approval, see Declarations.

### Instruments for diagnostic interviews

The M.I.N.I. (Mini International Neuropsychiatric Interview) (Sheehan D V: M.I.N.I. Mini International Neuropsykiatrisk Intervju för DSM-5, version 7.0.1. Swedish version, unpublished) was used to assess a number of psychiatric conditions, i.e., depression, suicidality, mania, panic disorder, agoraphobia, social anxiety, obsessive–compulsive disorder (OCD), post-traumatic stress disorder (PTSD), psychotic disorders, eating disorders, generalised anxiety disorder (GAD) in separate modules, all according to DSM-5. The new M.I.N.I. version 7.0.1. was chosen, since it also includes modules on antisocial personality disorder (ASPD), ADHD and attention deficit disorder (ADD). The M.I.N.I. also has modules addressing SUDs, but these were replaced by another instrument (see below). M.I.N.I. is structured in a similar way as SCID (Structured Clinical Interview for the DSM), i.e., each module starts with two initial questions. If one of these are given a positive response, they function as a key to open the module with all its questions. But if the initial questions are responded negatively, the interviewer goes on to the next module. M.I.N.I. can be performed in a much shorter time than SCID due to having fewer control questions. It was chosen since it is more feasible for telephone interviews and was deemed suitable for an epidemiologic study [29]. To validate a previous version of M.I.N.I., SCID-1 was used as golden standard and agreement between the two instruments was studied [30]. Cohen's kappa exceeded 0.50 for all diagnoses except for drug dependence, including above 0.70 for depression, panic disorder, agoraphobia, GAD, psychotic disorder, anorexia, bulimia, and PTSD. Sensitivity and specificity as well as positive and negative predictive values were good or very good for most diagnoses, but lower for drug dependence. Inter-assessor reliability was consistently high (К = 0.79–1.00). Test-retests varied more (К = 0.35–1.00) where mania and simple phobias accounted for the lower values. A Norwegian study [31] showed moderate test–retest reliability for hypomania, dysthymia, GAD, OCD, mood syndrome with psychosis, and alcohol abuse, but otherwise high values. Swedish validation is still lacking.

ADDIS (Alkohol Drog Diagnos Instrument) [32] was chosen to assess harmful use and dependence on 11 groups of substances (alcohol, sedatives/anxiolytics, opioids, cannabinoids, cocaine, central stimulants, ecstasy, hallucinogens, solvents, other drugs, and mixed drugs). ADDIS is the Swedish version of the American SUDDS [33]. ADDIS can be used for assessment according to DSM-IV, DSM-5 as well as ICD-10. Here the latter was chosen, since the Swedish health care applies the concept of substance dependence based on ICD-10. The original American SUDDS showed good agreement with experienced physician diagnoses, perfect specificity, and almost perfect sensitivity, while test-retests showed high global consistency [33, 34]. The internal consistency was satisfactory and similar in various ethnic groups. Swedish ADDIS showed construct validity for alcohol problems in a clinical as well as in a convicted DWI population [35]. The study also showed excellent discriminatory validity, and satisfactory to excellent internal consistency for the two populations, and for women, when analysed separately. ADDIS and SCID-1 were compared with regard to alcohol and drug problems with a golden standard (GS) based on all available documentation in individual cases [36]. ADDIS and SCID-1 showed high agreement with each other, although ADDIS had higher sensitivity than SCID-1. Agreement between ADDIS and GS was substantial to perfect for both alcohol and drugs, both currently and in lifetime. Sensitivity and specificity were substantial to perfect. Finally, ADDIS’ reliability was tested separately for all types of substances [37]. Internal consistency was excellent for all substance types on item level (all α > 0.95) as well as on criterion level (mean α = 0.93). Test–retest showed almost perfect systematic correlation for diagnostic evaluation concerning all substance types. The youth version, ADDIS-Ung, with wording of some questions adapted to adolescents, was used here. A validation study on ADDIS-Ung is in progress but not yet ready. In the present sample, however, internal consistency concerning lifetime dependence could be estimated (due to variance in all items) for eight specific substance groups (all but solvents) with α varying from 0.64 to 0.95 (mean α = 0.80), and concerning harmful use of alcohol, α = 0.65.

The instrument used to assess gambling problems was the National Opinion Research Center DSM-IV Screen for Gambling Problems (NODS). NODS demonstrated excellent test–retest reliability [38]. The validity and psychometric quality of NODS was also studied [39]. NODS demonstrated satisfactory internal consistency (α = 0.88). Concurrent validity was shown as high correlation with the South Oaks Gambling Screen, an instrument designed to assess gambling problems according to DSM-III. NODS was non-related to Addiction Severity Index composite scores on medical problems, indicating that gambling problems are measured well separated from other health issues. NODS scores 1–2 are interpreted as risky gambling, 3–4 as problem gambling and 5+ as pathological gambling [38].

A more recent diagnostic entity is the gaming disorder, i.e., the addictive behaviour related to video games. Conceptually introduced as a tentative diagnosis in the working process behind DSM-5, the gaming disorder was established as a manifest addictive diagnosis in the ICD-11. Here, IGDS (Internet Gaming Disorder Scale) [40] was used to assess gaming problems. A study on measurement quality in the UK, USA and Australia showed satisfactory to excellent internal consistency (α = 0.89–0.91) and confirmative factor analysis confirmed the one-factor solution [41]. IGDS is validated in a number of other countries (e.g. Portugal, Italy, Lebanon and Slovenia) indicating that it can be used internationally. IGDS has nine questions, answered with a five-grade Likert scale (with replies from “never” to “very often”), e.g. “Have you lost interests in previous hobbies and other entertainment activities as a result of your engagement with the game?” It results in scores of 9–45, where 17–20 is interpreted as risky gaming, while 21+ is interpreted as pathological gaming [42].

### Instruments for screening

As screening instruments on substance use problems, the Alcohol Use Disorders Identification Test (AUDIT) and the Drug Use Disorders Identification Test (DUDIT) were chosen [43, 44]. The total AUDIT scale varies from 0 to 40. Barbor [43] recommended the same cut-off (8 points) for men and women, while Bergman et al*.* [45] recommended eight points for men and six points for women. To increase sensitivity, we used a cut-off of six points for both genders. The total DUDIT scale varies from 0 to 44. An inclusive cut-off of two points was chosen. In addition to these, a scale constructed for LoRDIA called Substance Use Related Negative Consequences (SURNC) [46] was also used. It is based on questions on events in connection with drinking alcohol during the last year, with seven examples (e.g., Got into a fight, Injured yourself or someone else, Lost money or other valuables). The 90^th^ percentile (five points) was chosen as the cut-off of SURNC. All indicator scales had satisfactory internal consistency (AUDIT: α = 0.80; DUDIT: α = 0.78; SURNC: α = 0.77). A response above cut-off on any of the three led to invitation to an ADDIS interview.

As a screening instrument on psychiatric problems, the MINI Screen, version 7.0.0 [47], was chosen. It has in total 24 questions, designed to indicate 12 psychiatric syndromes, all of which are included in the M.I.N.I. Here only 22 questions were used, with the exclusion of alcohol and drug problems, since those were handled as mentioned. Since the MINI Screen did not include questions on ASPD and ADHD/ADD, although modules of these are included in M.I.N.I. 7.0.1., additional variables were used. Concerning ASPD, a scale on Delinquent behaviours [48] was used. To screen for ADHD/ADD, we used a question “Do you have the following functional disorders?” with separate answers (yes or no) to a number of specified disorders, including ADHD/ADD. Self-reported as ADHD/ADD led to invitation to interview. Although the MINI Screen includes indicators of various anxiety and mood problems, a scale related to emotions in general was also used, i.e., Psychosomatic problems (PSP) [49]. For Delinquent behaviours and PSP, the 90^th^ most severe percentiles were used as cut-offs; scoring above the 90^th^ percentile on either scale led to invitation to a M.I.N.I. interview. Both scales had satisfactory internal consistency (PSP: α = 0.88; Delinquent behaviours: α = 0.83).

For gambling, two questions were used: “Did you ever gamble for money? (Games where you bet money to win money, e.g.*,* games at casino, sports, lottery tickets, etc.)” and “Did you ever pay money within a computer game or mobile game?” For any of the two questions, the reply “yes” would lead to an invitation to a NODS interview.

For gaming, one question was used: “How many hours do you play computer games or video games (or similar on a mobile phone) on a normal weekday?” As a cut-off, “3–4 hours a day or more” was chosen for invitation to an IGDS interview.

### Procedure

As mentioned above, the screening instruments (except for gambling and gaming) were included in the questionnaires in Wave 5, whereas the indicators on gambling and gaming had not been decided when the Wave 5 data collection started with the first cohort. In the second cohort, all who participated in Wave 5 were screened for these problems. For the first cohort, however, the interviewers were instructed to screen for gambling and gaming on those who were contacted to be interviewed with M.I.N.I. or ADDIS, and if that screening then indicated that they scored above the cut-off, these responders would also be interviewed with NODS and IGDS, respectively.

Twenty trained interviewers made telephone contact after school with those who had scored above the cut-off on the screenings, informed them about the study, its purpose, voluntary participation, and confidentiality, while reminding them that they would be given a digital cinema ticket as a sign of gratitude if they agreed to complete the interview. Contact was made primarily by telephone. In the absence of a correct number, such was searched for by available web applications. If the adolescent could not be reached, the interviewer would try again up to about 10 times.

The interview concerned the problems of which screening was indicative, i.e., ADDIS was used if any of the substance use problems scored above the cut-off, NODS if the adolescents had acknowledged gambling for money, IGDS if they scored above the cut-off on gaming. Concerning other psychiatric problems, we tried to reduce the time for M.I.N.I. interviews to increase compliance in completing it. In clinical practice, all modules of M.I.N.I. are usually tried. For the telephone interviews, a shorter procedure was decided after consulting the creator of the instrument, David Sheehan. For most interviews, only the specific M.I.N.I. modules for the syndromes that were indicated from the screening were used. But in more complex cases, i.e., when more than three modules were indicated, the complete M.I.N.I. with all its modules was conducted.

### Estimations of prevalence and comorbidity

When estimating prevalence among all 949 Wave 5 participants, we assumed no diagnosis for those without screening indication of the addressed problem. We also assumed that among those selected for a particular interview (or a particular module in M.I.N.I.), the prevalence among those not interviewed (not reached or unwilling) would be the same as among those interviewed, since all had screening outcomes above the cut-off. Prevalence was estimated separately for girls and boys. The combined estimate is the mean of the two, thereby adjusting for differences in sample size between genders.

Estimated prevalence is given for the two most frequent substances, i.e. alcohol and cannabis, and combined for drugs other than these two. In addition, the prevalence of all substances combined is provided under “any substance”. Cases with sub-threshold indications are reported.

As for comorbidity, those who scored below the cut-off in one of the screenings for two interviews were assumed not to be comorbid in that particular dyad. Those (relatively few) who according to the screening should have been interviewed with both instruments but were not, were assumed to have the same distribution as those who were. The estimated percentages of comorbidity between types of problem were used to calculate the percentage of those with one type likely to have another type. These percentages were calculated as c/p, when c was the comorbidity rate from Table 5 below and p was the prevalence from Tables 2,3,4. For gaming and gambling, the observed comorbid cases were too few for these calculations to be reliable. We chose at least 12 observed comorbidity cases as our rule of thumb for conducting the calculations. Comorbidity as dyads of specified diagnostic entities were also analysed with Pearson Phi, tested for significance (Table 6) and interpreted as follows: Phi > 0.30 was regarded as substantial, > 0.20 as considerable, and below that – if significant – was regarded as moderate. For specific dyads, the number of cases decreases dramatically also concerning substance use and other psychiatric disorders, which is why genders then had to be combined to meet our rule of thumb of at least 12 comorbid cases for conducting the calculations.

### Study population

The total LoRDIA population are 1885 adolescents (928 girls, 957 boys) who chose to participate in waves 1 or 2 with their parents’ consent. This study, however, is based on those 949 adolescents (528 girls, 421 boys) who participated in Wave 5 in which the screening questions were included. In general, it was more problematic to collect data when the adolescents moved to senior high school. As could be noticed, participants in Wave 5 were more often girls (55.6%) than boys (44.4%). In Wave 5, the cohorts were about 17 years old, and the interviews in Wave 6 took place about one year later, when they were all about 18 years old.

### Representativeness of participants in Waves 5

Since Wave 5 included just about half of the LoRDIA population, participants and non-participants were compared as to gender, foreign origin, and family economy (Table 1). In addition, they were compared concerning demography, socioeconomic factors, as well as concerning emotional health (PSP), substance use involvement (SURNC) and delinquent behaviours based on questionnaire replies in Wave 3. From Wave 3, comparison was also done for an additional scale on emotional status measuring wellbeing, Psychological Health [50].

**Table 1:** Participants in screening (Wave 5) compared with non-participants

	*n*	Non-participants	Participants	*P*
n	1885	937	948	-
Gender, %	1885			< 0.001 (c)
girls	928	42.7	55.6	
boys	957	57.3	44.4	
Origin (a), %	1885			0.188 (c)
foreign	496	27.7	25.0	
Swedish	1389	72.3	75.0	
Household in relative poverty (b), %	1835			0.128 (c)
yes	71	4.6	3.2	
no	1764	95.4	96.8	
Psychological Health Scale in W3, m (sd)	1320	10.1 (1.77)	10.2 (1.65)	0.261 (d)
Psychosomatic problems (PSP) in W3, m (sd)	1290	18.0 (7.23)	18.0 (6.57)	0.854 (d)
Delinquent behaviours in W3, m (sd)	1295	1.02 (3.56)	0.55 (2.36)	0.007 (d)
Negative consequences (SURNC) in W3, m (sd)	1182	0.39 (1.52)	0.25 (1.11)	0.085 (d)

Notes: a) Born abroad or both parents born abroad; b) Household income below 60% of the median income based on tax registry data, c) Chi-2; d) T-test for independent groups

Those who participated in screening (Wave 5) did not differ significantly from non-participants in terms of foreign origin, family economy, psychological health, or psychosomatic problems. They did, however, differ in the following respects. Participants were more often female. They also were less involved in delinquent behaviours and had marginally fewer negative consequences of alcohol or drug use.

## Results

From the screenings, 758 adolescents (432 girls, 326 boys) scored above the cut-off on at least one of the indicators and were selected for an interview with at least one of the four instruments. However, only 387 (237 girls, 150 boys) were interviewed, i.e., 51.1% of those selected (54.9% of selected girls, 46.0% of selected boys).

Reasons for non-participation varied. In all, 243 persons (121 girls, 122 boys) could not be reached after several attempts, including 14 who had not provided a contact address, while 195 (104 girls, 91 boys) decided to decline participation once they had been reached. For six persons (three girls, three boys), information about these circumstances were not noted. In the following, we will address the outcomes of the four specific interviews separately.

### Substance use disorders

With scorings above cut-offs, a total of 273 were selected for an ADDIS interview (148 girls, 125 boys). Complete ADDIS interviews were conducted with 156 adolescents (100 girls, 56 boys). Alcohol dependence was found in 51 adolescents (36 girls, 15 boys) and harmful alcohol use in 23 adolescents (16 girls, 7 boys). Another 57 adolescents (31 girls, 26 boys) had 1–2 criteria on alcohol dependence, *i.e.* sub-threshold indications of a diagnosis.

The number of cases of SUDs (D = dependence, H = harmful use) and sub-threshold indications (ST) for other substances were: cannabis (D:9, H:2, ST:16), ecstasy (D:3, H:3, ST:3), cocaine (D:1, H:1, ST:1), sedatives/anxiolytics (D:1, ST:1), volatile solvents (D:1, ST:11), central stimulants (H:2, ST:4), opioids (H:1, ST:4), hallucinogens (H:1, ST:4), other drugs (ST:2), and mixed drugs (D:1, ST:7). When all substances are combined into “any substance”, there were 58 adolescents who developed any substance dependence (40 girls, 18 boys), and another 20 (14 girls, six boys) with harmful use, while 55 (31 girls, 24 boys) had sub-threshold indications of a SUD. Five girls and seven boys met the diagnostic criteria of multiple SUDs (with seven adolescents having two, three having three, and two having more than four SUDs). Based on the assumptions mentioned, the prevalence was estimated as shown in Table 2. We limit the presentation to lifetime diagnoses. Since few had time to enter "remission" from a previous diagnosis at an earlier age, for most, these diagnoses also represent the past year. In fact, none of those with lifetime dependence on alcohol or any of the drugs were in remission past year, but some cases with harmful use went into remission.^1^

**Table 2:** Outcomes of diagnostic interviews (numbers) with female and male adolescents, 18 years old, using ADDIS, and estimates of prevalence (%) of SUDs based on screenings and interviews combined

	Female adolescents			Male adolescents			Both genders
Lifetime diagnoses	Of interviewed in Wave 6 (*n* = 100)	Estimated (a) of all screened in Wave 5 (*n* = 528), including those lost to interview (LtInt)		Of interviewed in Wave 6 (*n* = 56)	Estimated (a) of all screened in Wave 5 (*n* = 421), including those lost to interview (LtInt)		Estimated (a) of all screened in Wave 5 (*n* = 949), including those lost to interview (LtInt)
	Assessment outcome	LtInt	**Prevalence, %**	Assessment outcome	LtInt	**Prevalence, %**	**Total prevalence, %** **Gender-adjusted (b)**
Alcohol		67			57		
Dependence	36		**10.4**	15		**7.1**	**8.8**
Harmful use	16		**5.1**	7		**3.3**	**4.2**
Sub-threshold	31		**11.4**	26		**12.4**	**11.9**
Cannabis		16			13		
Dependence	1		**0.9**	5		**1.4**	**1.2**
Harmful use	4		**0.1**	1		**0.2**	**0.2**
Sub-threshold	8		**1.7**	8		**2.4**	**2.1**
Other substances (c)		16			13		
Dependence	3		**0.6**	2		**0.5**	**0.6**
Harmful use	0		**0**	3		**0.7**	**0.4**
Sub-threshold	10		**2.1**	12		**3.1**	**2.6**
Any substance (d)		73			60		
Dependence	40		**13.1**	18		**8.8**	**10.9**
Harmful use	14		**4.5**	6		**2.9**	**3.7**
Sub-threshold	31		**10.2**	24		**11.9**	**11.1**

Notes: (a) Estimation is done with the assumption that screening below cut-off in Wave 5 can be interpreted as no diagnosis, and that those selected for but lost to interview have the same distribution as those interviewed. (b) Prevalence adjusted for different sample size of genders (mean of girls’ and boys’ prevalence). (c) Opioids, ecstasy, cocaine, central stimulants, sedatives/anxiolytics, volatile solvents, hallucinogens and “other”. (d) Persons with at least one of the kinds

In all, it was estimated that 14.6% of adolescents met the criteria of either dependence or harmful use of some substance. In that, alcohol dominated with 13% meeting the criteria of dependence or harmful use. We noticed that the prevalence of dependence and harmful use was higher among female adolescents compared to male adolescents. This pattern was consistent for alcohol and for all substances combined, but not for substances other than alcohol.

### Other psychiatric problems

A total of 766 adolescents (449 girls and 317 boys) were selected for M.I.N.I. interviews. M.I.N.I. interviews were conducted with 324 adolescents (206 girls and 118 boys). The M.I.N.I. provides both lifetime and current diagnoses for most but not all of the 16 assessed disorders, shown in Table 3.

**Table 3:** Outcomes of diagnostic interviews with female and male adolescents (numbers), 18 years old, using M.I.N.I., and estimates of prevalence of various psychiatric disorders (%) based on screenings and interviews combined

	Female adolescents			Male adolescents			Both genders
	Diagnosed among interviewed in Wave 6 (*n* = 237)	Estimated (a) of all screened in Wave 5 (*n* = 528), including those lost to interview (LtInt)		Diagnosed among interviewed in Wave 6 (*n* = 150)	Estimated (a) of all screened in Wave 5 (*n* = 421), including those lost to interview (LtInt)		Estimated (a) of all screened in Wave 5 (*n* = 949), including those lost to interview (LtInt)
	Assessment outcome	LtInt	**Prevalence, %**	Assessment outcome	LtInt	**Prevalence, %**	**Total prevalence, %** **Gender-adjusted (b)**
Depression		139			68		
Lifetime	72		**22.2**	19		**6.7**	**14.7**
Past year	19		**5.7**	5		**1.7**	**3.7**
Suicidal		100			55		
Lifetime	16		**4.4**	15		**4.9**	**4.7**
Past year	7		**1.9**	6		**1.9**	**1.9**
Manic episode		118			65		
Lifetime	6		**1.7**	6		**2.1**	**1.9**
Past year	1		**0.1**	2		**0.5**	**0.3**
Panic disorder		83			32		
Lifetime	42		**7.8**	8		**2.4**	**5.1**
Past year	12		**3.0**	3		**1.0**	**2.0**
Agoraphobia		84			32		
Past year	9		**2.3**	1		**0.2**	**1.3**
Social anxiety		91			35		
Past year	22		**5.7**	5		**1.2**	**3.5**
OCD		71			33		
Lifetime	22		**5.5**	9		**2.4**	**4.0**
PTSD		71			28		
Lifetime	2		**0.6**	0		**0**	**0.3**
GAD		84			30		
Lifetime	8		**2.1**	2		**0.4**	**1.3**
Psychotic syndrome		76			38		
Lifetime	10		**2.4**	10		**3.1**	**2.8**
Past year	1		**0.2**	2		**0.7**	**0.5**
Affective psychosis		76			38		
Lifetime	2		**0.6**	0		**0**	**0.3**
Past year	0		**0**	0		**0**	**0**
Anorexia		82			45		
Past year	2		**0.6**	0		**0**	**0.3**
Bulimia		75			28		
Past year	3		**0.8**	0		**0**	**0.4**
ASPD	2	97	**0.6**	3	66	**1.0**	**0.8**
ADHD	6	88	**1.5**	4	50	**1.2**	**1.6**
ADD	6	88	**1.5**	1	50	**0.5**	**1.0**
Any of the above psychiatric disorders	107	189	**36.4**	39	137	**17.0**	**26.7**

Notes: (a) Estimation of prevalence is done with the assumption that screening below cut-off in Wave 5 can be interpreted as no diagnosis, and that those selected for but lost to interview have the same distribution as those interviewed. (b) Prevalence adjusted for different sample sizes of genders (mean of girls’ and boys’ prevalence)

More than one in four adolescents (26.7%) were assessed as having a psychiatric disorder according to M.I.N.I., and these problems were more than twice as frequent in girls compared to boys (36.4% vs. 17.0%). This elevated prevalence in girls concerned depression and most anxiety disorders, e.g., panic disorder, agoraphobia, social anxiety, OCD, and GAD. Only very few met the criteria for PTSD, affective psychosis, anorexia, and bulimia, but they were all girls. Eighty of the adolescents, 59 girls (11.2%) and 21 boys (5%), met the criteria for more than one psychiatric disorder, ranging from two disorders (40 persons), three (20 persons), four (9 persons) and five to eight disorders (11 persons). For 50 of these (63%), this included a combination of depression and some type of anxiety disorder.

### Gambling and gaming problems

Screenings for gambling and gaming problems were not included in the Wave 5 questionnaires for Cohort 1. Still, 180 of the Cohort 1 participants (120 girls, 60 boys) were screened in connection with their ADDIS or M.I.N.I. interviews. For Cohort 2, all 489 Wave 5 participants were screened (242 girls, 247 boys). The number of screened adolescents for gambling and gaming persons therefore totalled 669 (362 girls and 307 boys). Based on screening, 254 were selected for an interview with NODS on gambling, while 100 were selected for an interview with IGDS on gaming. Looking at Cohort 2, we found that those who had been interviewed with ADDIS or M.I.N.I. had higher rates on gambling and gaming than those not interviewed. We adjusted for this selectivity in Cohort 1, by assuming that those not interviewed with ADDIS or M.I.N.I., and thus not screened in Cohort 1, would have the same distribution of gambling and gaming problems as those not interviewed with ADDIS or M.I.N.I. in Cohort 2. Estimations of prevalence of gambling and gaming are shown in Table 4.

**Table 4:** Outcomes of assessment (numbers) with NODS and IGDS for girls and boys, 18 years old, and estimates of prevalence (%) of at risk and problem behaviour of gambling and gaming, based on screenings and interviews combined

	Female adolescents			Male adolescents			Both genders
	Assessed in Wave 6	Estimated (a, b) of all in Wave 5 including lost to screening (LtScr = 286) or to interview (LtInt),		Assessed in Wave 6	Estimated (a, b) of all in Wave 5 including lost to screening (LtScr = 174) or to interview (LtInt)		Estimated (a, b) of all in Wave 5 including lost to screening (LtScr) or to interview (LtInt)
	Assessment Outcome	LtInt	Prevalence, %	Assessment Outcome	LtInt	Prevalence, %	Prevalence, % Gender-adjusted (c)
	*n* NODS = 43 *n* IGDS = 8		*n* = 528	*n* NODS = 82 *n* IGDS = 29		*n* = 421	*n* = 949
Gambling		23			106		
Pathological Problem gambling At risk	1 0 3		**0.4** **0** **0.7**	5 7 20		**2.4** **3.4** **9.7**	**1.4** **1.7** **5.2**
Gaming		14			49		
Pathological At risk	0 1		**0** **0.2**	6 11		**2.3** **5.7**	**1.2** **3.0**

Notes: (a) Estimation is done with the assumption that screening below cut-off in Wave 5 can be interpreted as no problem or risky behaviour, and that those selected for but lost to interview have the same distribution of risk and problem behaviours as those selected and interviewed. (b) Those not screened in Cohort A (166 women, 114 men), due to not being interviewed with ADDIS or M.I.N.I., were assumed to have the same distribution as those in Cohort B who also were not interviewed with ADDIS or M.I.N.I. Prevalence therefore depends on the findings per cohort, which means prevalence figures are not directly related to the total number of observed cases. (c) Prevalence adjusted for different sample size of genders (mean of girls’ and boys’ prevalence)

The estimation of total prevalence of gamblers at risk was 5.2% and of problem gamblers 1.7% and pathological gamblers 1.4%, while the total prevalence of gamers at risk was 3.0% and pathological gamers 1.2%. Both these problems were mostly found among boys, and not among girls. Although there were some variations between cohorts, these gender differences were stable between cohorts.

### Comorbidity – estimated prevalence

Comorbidity between SUDs, other psychiatric disorders, and gambling and gaming problems was investigated as the lifetime occurrence of these problems in pairs (Table 5). For SUDs, dependence and harmful use of any substances were combined (not sub-threshold), for gambling problems, pathological and problem levels were included (not at risk), and for gaming only pathological levels (not at risk) since gaming has no problem level defined. Other psychiatric problems were collapsed into one measure, i.e., psychiatric disorder.

**Table 5:** Estimated prevalence of comorbidity (%, *n* = 949) between four types of problems: substance use and psychiatric disorders, as well as gambling and gaming problems

	Psychiatric disorder	Gambling problem	Gaming problem
SUD			
Girls	7.0%	0%	0%
Boys	2.6%	2.6%	2.0%
Total	4.8%	1.3%	1.0%
Psychiatric disorder			
Girls		0.3%	0%
Boys		3.3%	1.3%
Total		1.8%	0.7%
Gambling problem			
Girls			0%
Boys			0.9%
Total			0.4%

The highest comorbidity involved SUD and psychiatric disorders, with a total of 4.8% being comorbid, dominated by females (7.0%). All other comorbidity pairs of problems were dominated by males, although at low levels. The highest type of comorbidity among boys involved psychiatric disorder vs. gambling problems (3.3%), followed by SUD vs. psychiatric disorders and vs. gambling problems (both 2.6%).

Calculating the percentage of those with one type likely to have another type shows that about 40% of girls with a SUD also had a psychiatric disorder, while on the other hand more than 28% of girls with a psychiatric disorder also had a SUD. In boys with a SUD, 22% had another psychiatric disorder, while 15% of those with a psychiatric disorder also had a SUD. Estimations of these comorbidities in gamers and gamblers were not calculated due to low numbers.^2^

### Comorbidity – specified dyads

We also investigated specified comorbidities, i.e., the rate found in specified dyads of the ten most frequent disorders. This investigation only included cases with confirmed information from screening and interview assessment, i.e., not estimates for non-interviewed. Specified comorbidities were assessed with Pearson Phi, tested for significance. Genders were combined to facilitate testing. The Phi statistic, p-value, and number of comorbid cases in each dyad are shown in Table 6.

**Table 6:** Psychiatric comorbidity between disorders with at least 12 observed cases (a). Pearson Phi, tested for significance (p) and dyadic observation numbers (paired n)

Phi P Paired n	2 Drug use disorder (DUD)	3 Gambling problem	4 Depression	5 Suicidality	6 Panic disorder	7 Social anxiety	8 OCD	9 Psychotic syndrome	10 ADHD/ ADD (c)
1. Alcohol use disorder (AUD)	0.287 0.000 816	0.076 0.088 508	0.143 0.000 696	0.087 0.018 735	0.040 0.276 754	0.107 0.003 738	0.044 0.225 756	0.085 0.019 761	0.088 0.125 749
2. Drug use disorder (DUD) (b)		0.054 0.218 528	0.129 0.000 733	0.071 0.046 781	0.045 0.199 816	0.083 0.018 805	-0.026 0.455 823	0.040 0.251 818	0.108 0.002 796
3. Gambling problem			0.154 0.001 484	0.115 0.010 503	0.072 0.101 513	0.132 0.003 506	0.115 0.010 507	0.224 0.000 516	-0.031 0.485 511
4. Depression				0.228 0.000 722	0.440 0.000 735	0.374 0.000 713	0.269 0.000 719	0.216 0.000 728	0.180 0.000 718
5. Suicidality					0.295 0.000 777	0.107 0.003 747	0.260 0.000 765	0.175 0.000 780	0.187 0.000 763
6. Panic disorder						0.330 0.000 780	0.376 0.000 788	0.158 0.000 815	0.204 0.000 796
7. Social anxiety							0.255 0.000 784	0.196 0.000 772	0.161 0.000 7 55
8. OCD								0.258 0.000 790	0.187 0.000 770
9. Psychotic syndrome									0.084 0.018 796

Notes: a) Disorders excluded here due to less than 12 cases: Gaming, Manic episode, Agoraphobia, PTSD, Affective psychosis, Anorexia, Bulimia, GAD, ASPD. b) All assessed SUDs other than AUD are collapsed to DUD. c) ADHD and ADD are here combined

Substantial comorbidity (Phi > 0.30) was found between depression and panic disorder, depression and social anxiety, panic disorder and social anxiety, and panic disorder and OCD.

Considerable comorbidity (0.30 > Phi > 0.20) was found between AUD and DUD, gambling and psychotic disorder, depression and suicidality, depression and OCD, depression and psychotic syndrome, suicidality and panic disorder, suicidality and OCD, panic disorder and ADHD/ADD, social anxiety and OCD, and between OCD and psychotic syndrome.

Moderate comorbidity (Phi < 0.20, but significant) was found between AUD and depression, AUD and suicidality, AUD and social anxiety, AUD and psychotic syndrome, DUD and depression, DUD and suicidality, DUD and social anxiety, DUD and ADHD/ADD, gambling and depression, gambling and suicidality, gambling and psychotic syndrome, gambling and OCD, depression and ADHD/ADD, suicidality and social anxiety, suicidality and psychotic syndrome, suicidality and ADHD/ADD, panic disorder and psychotic syndrome, social anxiety and psychotic syndrome, social anxiety and ADHD/ADD, OCD and ADHD/ADD, and between psychotic syndrome and ADHD/ADD.

## Discussion

The present study is one of few describing the prevalence and comorbidity of mental health disorders in the young general population, and which cover a broad range of psychiatric disorders, including both SUDs and gambling- and gaming-related diagnoses. Comorbid mental health disorders were common in individuals who fulfilled the criteria of SUDs, and vice versa, with common comorbid SUDs in those who met the criteria of other psychiatric disorders. Such mental health comorbidity was more pronounced in girls than in boys.

In contrast with previous national survey data on adults [14], SUDs tended here to be more common in young female participants than in their young male counterparts. This was also found in the youngest group (17–29 years) of a national, general population survey used for comparison [14], although differences were not as pronounced as here. The same picture has been seen in adolescents in clinical treatment settings; despite the male predominance in alcohol-related diagnoses in the adult general population, these diagnostic entities in the present dataset were at least not more common in boys in child and adolescent emergency psychiatry [51].

In addition to those for which we could establish SUD diagnoses (dependence or harmful use), we also found adolescents who met one or two criteria for substance dependence but without the three criteria that are required for a diagnosis, and at the same time not meeting the criteria for harmful use. Such undiagnosed sub-threshold cases in ICD-10 or DSM-IV have been referred to as “diagnostic orphans” [52] and have been found to represent a significant proportion of adolescent drinking populations, about as many as those dependent [53, 54]. As for AUD according to DSM-IV, diagnostic orphans were compared to other users without a diagnosis and found to drink more and use more cannabis or other drugs [52]. Their patterns were more like those with alcohol abuse, possibly indicating that these might be in a process of developing substance dependence. As we saw, the great majority of those with subthreshold criteria as well as of those with harmful use for most drugs showed stability in the SUD with recent symptoms past year. Concerning dependence, none were in remission.

Mental health comorbidity was markedly more common in girls with a SUD than in boys with a SUD. This is consistent with previous literature showing that the association between some of the more common mental health problems, anxiety and depression, and the association between such symptoms and alcohol-related behaviour is stronger during adolescence in girls than in boys [55, 56]. Thus, the heightened comorbidity with these conditions in the present study in girls is in line with existing data. Likewise, ADHD has been shown to be a stronger risk factor for alcohol-related diagnoses in girls than in boys. Both in adolescents with a clinical diagnosis and in those without a clinical diagnosis, but who screen positive for ADHD, the association to alcohol problems has been shown to be stronger in girls [57]. In girls and boys treated for SUDs in out-patient facilities, the picture has been clearer; self-reported mental health symptoms, as well as self-reported mental health disorders diagnosed, were significantly more common in girls than in boys [58].

Prevalence of ADHD and ADD were on comparable levels compared to previous studies, possibly lower, and not higher in girls than in boys. ADHD is a risk factor for SUD in the young [59]. Here, it should be borne in mind that participants in the present study, compared to those who dropped out of the cohort, had lower scorings on externalising behaviour problems, and that the prevalence of ADHD can be suspected to be slightly too low in this sample because of that. ADHD can be found in around 25 percent of adolescents in treatment for SUD [60], and research has pointed to the importance of screening for comorbid ADHD and substance use in adolescents in clinical settings [61]. Importantly, the combination of these conditions is known to increase the severity of the clinical condition, and to present a challenge in diagnostic and therapeutic interventions [61].

Specified comorbidities, i.e. dyads of specified disorders, could be calculated for the ten most frequent disorders (although not separately for genders). Out of the 45 dyads surveyed, comorbidity was found in 35, i.e., in just over 3/4 of the dyads. Depression, suicidality and social anxiety are each listed for maximum possible comorbidities, i.e. nine out of nine. Psychotic syndrome is listed for eight comorbidities, OCD and ADHD/ADD for seven, panic disorder for six, while AUD, DUD and gambling are listed for five comorbidities each. Therefore, the psychiatric disorders show greater variation in comorbidities compared to SUDs and gambling.

The correlations between each of the conditions assessed here were generally low, with the clear exception of the correlation between AUD and DUD. In contrast, gambling was markedly less associated with SUD. For gambling, correlations were instead stronger with psychiatric disorders. This may seem to suggest a somewhat different role of gambling behaviour in adolescents, in some contrast to the role of alcohol and drugs in the young age groups, as also suggested in some previous Swedish data from young adults [62].

A comparison of comorbidity in boys and girls could not be carried out in behavioural addictions, i.e., in individuals with problematic gambling and gaming patterns, because of the low absolute number of problem gamblers or problem gamers among girls. Thus, larger studies in the area are needed. Here, the pre-existing literature is consistent with the findings from the SUD area, such that comorbid mental health disorders are more common in female problem gamblers than in their male counterparts [63]. Meanwhile, for problem gaming and gaming disorders, the literature on mental health comorbidity is markedly less extensive, and there is need for studies to address problem gaming and comorbidity in larger population samples. Rates of problem gambling in the present study were found to be around three percent, when collapsing the data for the actual disorder with those having a gambling problem below diagnostic level. These figures are comparable to those reported in previous studies. In a general population survey in Sweden, within the 16–24-year age group, the prevalence of problem gambling has been reported to be seven percent in young men and around 1.5 percent in young women [64]. In a different study, carried out as a six-year follow-up after a baseline high school survey when participants had reached 24 years, only two percent among these 24-year-olds screened positive for lifetime problem gambling, with a non-significant difference between genders [62]. In some contrast to these other studies, a recent study in adolescents in Sweden demonstrated a relatively high number of problem gamblers among boys (and with low figures in girls). Problem gambling in Swedish adolescents was detected in 12 percent of boys in ninth grade, compared to one percent in girls, and in 14 percent of boys in high school (second grade), compared to less than one percent in girls [65]. A previous study on boys aged 16 and 18 years found 16 percent to be pathological gamblers [12].

The prevalence of problem gamers overall has been insufficiently studied in the general population. In a web survey-based study in the Swedish general population, probably with a risk of oversampling of individuals with extensive online behaviours, participants were screened for gaming problems including likely addictive gaming, using a seven-item screening instrument. In the youngest age groups (15–18 years, and 19–24 years), three percent and one percent, respectively, were categorised as addicted gamers, and 15 and 11 percent as problem gamers, respectively [66]. In a more recent study addressing the general population, involving the age group ranging from 16 to 24 years [67], 11 percent were categorised as problem gamers or addicted gamers. Again, this type of online survey may cause an oversampling of individuals with gaming behaviours [68]. In a large Norwegian study sample of gamers recruited from the general population, around one percent were found to be addicted to gaming, while around seven percent were categorised as problem gamers [69]. Overall, rates of gaming disorder have demonstrated a substantial variability, and there is still a need for further prevalence studies of this relatively novel condition. In Steven’s and co-workers’ study, around two percent of populations fulfilled the criteria of a gaming disorder. Further studies are needed in order to highlight prevalence figures in different settings and with comparable diagnostic measures [68].

Both for gambling and gaming, very large gender differences were seen. Men are more likely than women to develop a gambling disorder, and a relatively consistent research finding is that women develop their gambling problems in a markedly later phase in life. While the severity and time course of gambling problems may be more problematic in women than in men [64], the disorder typically develops later in women, sometimes referred to as a ‘telescoping phenomenon’ [70, 71]. Thus, in an adolescent population, it is expected that women are less likely than men to have initiated gambling.

For gaming, few studies are available for comparison, including within the present Swedish setting. A general population survey in students aged around 16 and 18 [72], extensive gaming behaviours were around six times more common in boys than in girls. Likewise, in a prevalence study originating from two online surveys, boys were around 60 percent more likely than girls to be at least problem gamers, although absolute numbers were small and limit the conclusions to be drawn [66]. Likewise, a Norwegian study in gamblers demonstrated a clearly higher prevalence of problem gaming in men [69]. Globally, it has been estimated that men are around 2.5 times more likely than women to have a gaming disorder, although further studies are needed [68].

### Methodological discussion

This is the first Swedish study that has tried to estimate psychiatric prevalence and comorbidity in a community sample of adolescents based on structured interviews. There are of course reasons for the previous lack of such a study; for example, it is known to be very costly and hard to execute. As expected, we encountered some challenges.

The two-phase model, recommended by Dunn et al. [20] was deemed as the most practical way to carry out the study. As mentioned, however, we were not faithful to the model when it came to gambling and gaming in Cohort 1. Although the model seems to be the most feasible, a limitation is the risk of missing those who were healthy at the time of screening in Wave 5 but developed a disorder in the following year.

We chose an inclusive strategy in the first phase, with several complementary indicators to increase the likelihood that all positive cases would be included. We also applied lower cut-offs than is usually done. And indeed, this resulted in large numbers of persons selected for interview. In the second phase, the interviews were conducted by trained interviewers who also had professional and clinical experience in the field.

The interview instruments were selected based on both feasibility and quality requirements. Both ADDIS and M.I.N.I. are recommended by the National Board of Health and Welfare in its national guidelines [73]. Although SCID is often regarded as a “Golden standard” for psychiatric assessment, M.I.N.I. was chosen, since it can be performed in a shorter time and is more feasible for telephone interviewing [29]. However, clinical observations on psychosis (three items included in the K module) could not be done by telephone, and after advice from the creator of M.I.N.I., David Sheehan, we decided to leave these three items out. M.I.N.I. has been validated internationally but not yet in Swedish. In general, it has demonstrated good properties. Two modules on SUDs with some identified weaknesses [30, 31], were here replaced by ADDIS which has strong validation for all types of SUDs and is the only instrument to give specific diagnostic proposals for all types of substances [29]. The youth version, used here, is quite similar to the adult version, but not yet validated. A check on internal consistency in the present sample, showed acceptable to excellent alpha-values. NODS and IGDS have been validated internationally, although not in Sweden. Both these are relatively short screening instruments but are based on the DSM-5 proposals.

The greatest challenge, as expected, turned out to be the large loss of interviews with selected persons, due to difficulties in making contact or their unwillingness to participate. We estimated their outcomes based on the assumption that prevalence in those lost to interview was roughly comparable to those who were interviewed, since they all had screening outcomes over cut-off. But as screening does not capture diagnostic outcome, we urge some caution in interpreting our results. A similar problem concerned the lack of screening for gambling and gaming problems in Cohort 1. This was handled by using prevalence estimations of non-screened based on Cohort 2 data for a similar sub-population – those not selected for interviews with M.I.N.I. or ADDIS. Here too, some caution in interpreting the results is needed.

Another problem is that we had to start our two-phase strategy with the participants in Wave 5 representing about half of the LoRDIA study population. These were more often girls, a problem that was handled by using gender-separated analyses. In a previous screening (Wave 3), those participating in Wave 5 were less involved in delinquent behaviours and had marginally fewer negative consequences of substance use compared to non-participants. Thus, those lost to Wave 5 show more externalising problem behaviours. Overall, we conclude that certain aspects of comorbidity may be less sufficiently captured in our study. Therefore we must warn that the prevalence of ASPD and ADHD may be underestimated here, although ADHD and ADD were, as mentioned above, on the same level as in previous research. Our findings on these disorders may be seen as a first attempt, and we hope that this study will be followed by others.

Still, our findings present a much higher prevalence of general psychiatric ill health than the international reviews presented in our introduction [1, 3]. Both these, however, included children and adolescents of a much younger age (starting from five or six years), when many of these problems have not yet developed. The higher prevalence in our study of 18-year-olds should be expected since onset of SUDs as well as other psychiatric disorders is known to peak in mid-adolescence [26]. An exception was the prevalence of generalized anxiety disorder which in the present study was somewhat lower, although quite comparable to a previous study in the general adolescent US population [74].

### Implications

The key implications of the present findings include the structured screening and diagnostic routines in the area of substance use and mental health in the young, as well as the need to develop early, integrated treatment interventions for adolescents with combined conditions. Also, such efforts need to be particularly emphasised in young women; while SUD was overall higher in girls than in boys in the present study, its comorbidity figures were also higher than in boys.

### Conclusion

Substance use disorders are common in adolescents, and in a Swedish setting, alcohol predominates. However, mental health disorders are common in individuals with substance-related problems, and this fact calls for improved screening in patients with each of the conditions, and for integrated treatment approaches for individuals with comorbid conditions. As in previous studies, young women with substance use disorders are at higher risk of psychiatric comorbidity than their male counterparts. Gambling- and gaming-related disorders warrant further evaluations, including larger study samples for young women, where prevalence figures are markedly lower than among young men.

## Data Availability

The datasets generated and analysed in the current study are not publicly available due to confidentiality statutes of the Regional Research Review Board and of the Swedish Ethical Review Authority. The programme is still ongoing, but anonymised data sets with a limited number of chosen variables may be available from the corresponding author on reasonable request and with a fee. When the programme is eventually completed, totally anonymised data sets (secured against backward identification) will be publicly available in cooperation with the Swedish Data Services.

## Abbreviations

ADD: Attention deficit disorder
ADDIS: Alkohol Drog Diagnos InStrument [Alcohol Drug Diagnostic Instrument]
ADHD: Attention deficit hyperactivity disorder
ASPD: Antisocial personality disorder
AUD: Alcohol use disorder
AUDIT: Alcohol use disorders identification test
CAN: Centralförbundet för alkohol- och narkotikaupplysning [The Swedish Council for Information on Alcohol and Other Drugs]
DSM: Diagnostic and statistical manual of mental disorders
DUD: Drug use disorder
DUDIT: Drug use disorders identification test
DWI: Driving while intoxicated
GAD: Generalised anxiety disorder
ICD: International statistical classification of diseases and related health problems
IGDS: Internet gaming disorder scale
LoRDIA: Longitudinal Research on Development In Adolescence
M.I.N.I: Mini international neuropsychiatric interview
NODS: NORC DSM-IV Screen for Gambling Problems
NORC: National opinion research center
OCD: Obsessive–compulsive disorder
PSP: Psychosomatic problems scale
PTSD: Post-traumatic stress disorder
SCID: Structured clinical interview for the DSM
SOU: Statens Offentliga Utredningar [State public investigations]
SUD: Substance use disorder
SUDDS: Substance use disorder diagnostic schedule
SURNC: Substance use related negative consequences

## Footnotes

1 When genders are combined, the following had no longer harmful use past year: alcohol 2 cases, cannabis 1, central stimulants 1, ecstasy 2, and hallucinogens 1 case.  In addition, some cases who met subthreshold criteria lifetime had no symptoms past year: alcohol 6 cases, opioids 1, cannabis 3, central stimulants 2, ecstasy 1, hallucinogens 1, and other drug 1 case.

2 Observed cases of comorbidity (not estimated): IGDS vs. NODS 3 cases (male), IGDS vs. ADDIS 2 cases (male), NODS vs. ADDIS 4 cases (male), IGDS vs. M.I.N.I. 2 cases (male), NODS vs. M.I.N.I. 7 cases (1 female, 6 male).
